# Average reward rates enable motivational transfer across independent reinforcement learning tasks

**DOI:** 10.3389/fnbeh.2022.1041566

**Published:** 2022-11-09

**Authors:** Kristoffer C. Aberg, Rony Paz

**Affiliations:** Department of Brain Sciences, Weizmann Institute of Science, Rehovot, Israel

**Keywords:** reinforcement learning, average reward, motivation, prediction error, behavioral modeling, reward, transfer, controllability

## Abstract

Outcomes and feedbacks on performance may influence behavior beyond the context in which it was received, yet it remains unclear what neurobehavioral mechanisms may account for such lingering influences on behavior. The average reward rate (ARR) has been suggested to regulate motivated behavior, and was found to interact with dopamine-sensitive cognitive processes, such as vigilance and associative memory encoding. The ARR could therefore provide a bridge between independent tasks when these are performed in temporal proximity, such that the reward rate obtained in one task could influence performance in a second subsequent task. Reinforcement learning depends on the coding of prediction error signals by dopamine neurons and their downstream targets, in particular the nucleus accumbens. Because these brain regions also respond to changes in ARR, reinforcement learning may be vulnerable to changes in ARR. To test this hypothesis, we designed a novel paradigm in which participants (*n* = 245) performed two probabilistic reinforcement learning tasks presented in interleaved trials. The ARR was controlled by an “induction” task which provided feedback with a low (*p* = 0.58), a medium (*p* = 0.75), or a high probability of reward (*p* = 0.92), while the impact of ARR on reinforcement learning was tested by a second “reference” task with a constant reward probability (*p* = 0.75). We find that performance was significantly lower in the reference task when the induction task provided low reward probabilities (i.e., during low levels of ARR), as compared to the medium and high ARR conditions. Behavioral modeling further revealed that the influence of ARR is best described by models which accumulates average rewards (rather than average prediction errors), and where the ARR directly modulates the prediction error signal (rather than affecting learning rates or exploration). Our results demonstrate how affective information in one domain may transfer and affect motivated behavior in other domains. These findings are particularly relevant for understanding mood disorders, but may also inform abnormal behaviors attributed to dopamine dysfunction.

## Introduction

Negative information, such as bad news or negative feedback, may affect behavior in subsequent everyday-life tasks that are unrelated to the received information. Despite being relevant for understanding the impact of, for example, mood-related dysfunctions on cognitive performance, few studies investigated the neurocomputational correlates of how information received in one task may linger and affect behavior in other unrelated tasks.

One theory posits that motivated behavior depends on the average reward rate (ARR), such that vigilance and response rates increase in contexts were rewards are frequently provided (Niv, 2007; Niv et al., 2007). In support, human research report that response vigor (Guitart-Masip et al., 2011; Rigoli et al., 2016), as well as memory encoding success (Aberg et al., 2017, 2020), are affected by ARRs. Accordingly, the ARR may provide a bridge which enables recently received affective information (e.g., negative news or feedbacks) to temporally extend and influence subsequent motivational states and task performances.

The motivational impact of average reward levels was coupled with BOLD signal in a midbrain region pertaining to the loci of dopamine neurons (Aberg et al., 2020), while other studies report associations between ARRs and dopamine release in the nucleus accumbens (Hamid et al., 2016; Mohebi et al., 2019). Reinforcement learning depends on the neural representation of prediction errors (i.e., the mismatch between an actual and a predicted outcome; Sutton and Barto, 2018), which are coded by midbrain dopamine neurons and their downstream targets, including the nucleus accumbens (Rutledge et al., 2010; Hart et al., 2014; Schultz, 2016). Accordingly, reinforcement learning tasks may be particularly vulnerable to manipulations of average reward levels.

Moreover, average reward levels may affect learning performance *via* different computational mechanisms, e.g., *via* altered learning rates, decision biases, or reward processing, and this may be due to an accumulation of prediction errors (Eldar and Niv, 2015) or rewards (Aberg et al., 2020). To disentangle the impact of average reward on these different computational mechanisms, we designed and confronted a number of different behavioral models.

Here, participants performed two reinforcement learning tasks presented in interleaved trials. An “inducer” task was used to control the ARR by providing probabilistic rewards with a low, medium, or a high probability. A “reference” task, with a constant (medium) reward probability, was used to estimate the impact of ARRs on learning performance in the different conditions. In three separate experiments, we observed lower learning performance in the reference task when it was presented together with an inducer task that provided probabilistic rewards with a low probability (as compared to medium and high probabilities). Furthermore, careful behavioral modeling revealed that the impact of ARRs was best described by models which (i) accumulate average rewards (rather than prediction errors), and (ii) allow the ARR to directly modulate the prediction error signal (rather than learning rates or decision making biases).

Abnormal reinforcement learning patterns may play a role in the acquisition and maintenance of dysfunctional behaviors in relation to psychiatric and neurological disorders (Maia and Frank, 2011). Therefore, understanding how reinforcement learning is affected by transfer of affective information between tasks, and how these related to motivated behaviors and interact with the dopamine system, is relevant for psychopathology.

## Materials and methods

### Participants

The study followed the declaration of Helsinki and was approved by the Institutional Review Board (IRB) of the Weizmann Institute. Informed consent was provided before the start of the testing. In total, 245 participants were recruited *via* Amazon Mechanical Turk (*n* experiment 1/2/3 = 63/79/103). Inclusion criteria consisted of being older than 18 years, speaking English fluently, and having completed more than 95% of previously started assignments on the Amazon Mechanical Turk platform. Each participant could only perform one of the three experiments, and were recruited *via* identical ads on the Amazon Mechanical Turk platform, and their participation was decided on a first-come-first-served bases. Participants were excluded from the data analysis if their average overall performance on the last four trials was less than 0.6, if they failed to respond on more than 20 trials, or if they exhibited more than two response sequences where the same button was pressed more than ten times in a row. After applying these exclusion criteria, data from 148 participants were included in the analyses (*n* experiment 1/2/3 = 50/58/40).

### Task and procedure

**FIGURE 1:**
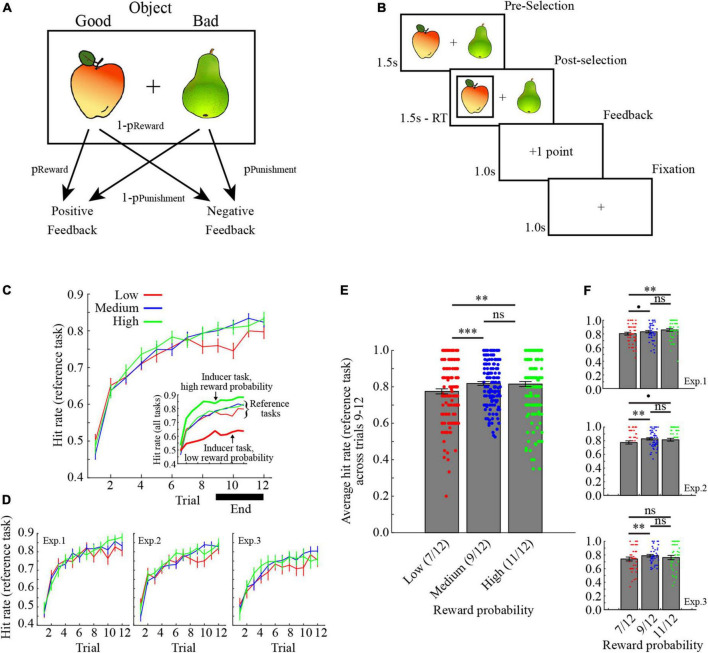
Task description and learning performance. **(A)** In each trial, participants selected one of two options in a pair. One object provided positive feedback with probability *p*_Reward_, while the other object provided negative feedback with probability *p*_Punishment_. Here, *p*_Reward_ was always equal to *p*_Punishment_. **(B)** Illustration of a trial. **(C)** Learning curves for the reference tasks, collapsed across the three experiments. The inset also shows performance for the inducer tasks with low- and high reward probabilities. Inducer/reference tasks are, respectively denoted by bold / narrow lines. **(D)** Learning curves for the reference task in the three experiments separately. **(E)** Average learning performance for the reference tasks across trials 9–12 collapsed across the three experiments. **(F)** Average learning performance for the reference tasks across trials 9–12 separately for the three experiments. ***p < 0.001, **p < 0.01, *p < 0.05, *p* < 0.10, ns *p* > 0.10. All error bars indicate the standard error of the mean.

Participants performed probabilistic reinforcement learning tasks, where in each trial one object in a pair of objects was selected (Figure 1A). Probabilistic feedback was then presented based on the reward/punishment probability assigned to the selected object. For example, as illustrated in Figure 1A, selecting the apple would yield positive feedback with probability *p*_*Reward*_, while selecting the pear would yield negative feedback with probability *p*_*Punishment*_. In the present study, *p*_*Reward*_ was always equal to *p*_*Punishment*_. Critically, to control the ARR, here defined as average rewards per trials, an “inducer” task was performed in interleaved trials together with a “reference” task. The “inducer” task provided positive feedback with *p*_*Reward*_ set to either high (11/12, *p*_*Reward*_∼0.92), medium (9/12, *p*_*Reward*_ = 0.75), or low (7/12, *p*_*Reward*_∼0.58), while for the “reference” task *p*_*Reward*_ was always set to medium (9/12, *p*_*Reward*_ = 0.75). In other words, the ARR was, respectively highest and lowest in conditions where the inducer task provided a high/low probability of reward.

Each trial started with a display of two images for a maximum of 1.5 s (Figure 1B). After selection, the selected object was marked for the remainder of the 1.5 s, followed by a feedback displayed for 1 s. A fixation cross was then displayed for 1 s before the start of the next trial. If participants failed to respond within 1.5 s, a screen displaying the sentence “Too Slow! –2 points” was displayed for 3 s.

Each task was performed for 12 trials in each block (for a total of 24 trials per block, i.e., 12 inducer task trials +12 reference task trials), and the order of trials for each task within a block was interleaved in a pseudorandom fashion, such that no task was repeated for more than three trials in a row (e.g., … Inducer-Reference-Reference-Inducer-Inducer … is correct, while … Inducer-Reference-Reference-Reference-Reference … is incorrect).

Each participant performed five blocks of trials for each of the three conditions (i.e., five blocks each with *p*_*Reward*_ of the inducer task set to high, medium, and low), for a total of 15 blocks and 360 trials. The order of blocks was pseudorandomized with the limitation that one block of each condition had been performed before repeating a condition (e.g., … Low-High-Medium, Low-Medium-High, Medium-High-Low … is a correct example of three possible sequential blocks, while … Low-Low-Medium, High-Low-Medium, High-High-Medium … is an example of an incorrect sequence of three blocks). In each task, a new pair of objects were presented for a total of 32 different pairs of objects (15 blocks of two interleaved tasks in the experiment proper +1 training block).

Before the start of the experiment, participants received explicit instructions on how to perform the task. For example, in experiment 1 the instructions were:

*“Two objects will be presented in each trial, one to the left and one to the right. Select the object on the left by pressing the ‘LeftArrow’ key and the object on the right by pressing the ‘RightArrow’ key. Selecting an object results in one of the following types of feedback:*

*+1: you gained 1 point.*

*−1: you lost 1 point.*

*Importantly! Collecting points will earn you a monetary bonus.*

*One object in each pair is more likely to give better feedback than the other.*

*Increase your bonus by learning which are the best objects.*

*Each pair of objects is presented for 1.5 s. Beware! If your response is too slow, you will lose 2 points.*

*Press the SPACE bar to start the task.”*

After having read the instructions, participants performed one training block before continuing to the experiment proper. The experiments lasted less than 30 min, and participants were paid $2 for their participations and up to $2 extra as a performance-based bonus.

#### Experiments

Three different experiments were conducted, all based on the procedure described above, but with different feedback values.

##### Experiment 1

Positive/negative feedbacks were, respectively +1/−1 points for both the inducer tasks and the reference task.

##### Experiment 2

Positive/negative feedbacks were set to +1/−1 points for the reference task, while positive/negative feedback was +0.6/−0.6 for the inducer task with high p_*Reward*_, +1/−1 for the inducer task with medium *p*_*Reward*_, and +3.0/−3.0 for the inducer task with low *p*_*Reward*_. These specific feedback values provided the same average number of points if the best option is selected in all trials (i.e., low *p*_*Reward*_: 7 × 3/12 + 5 × − 3/12 = 0.5 points/trial; medium *p*_*Reward*_: 9 × 1/12 + 3 × − 1/12 = 0.5 points/trial; high *p*_*Reward*_: 11 × 0.6/12 + 1 × − 0.6/12 = 0.5 points/trial).

##### Experiment 3

Positive/negative feedbacks were set to +1/0 points for both the inducer tasks and the reference task. This experiment is identical to experiment 1, except that the negative (−1 point) feedback was replaced by a neutral (0 point) feedback.

### Behavioral modeling

All models are based on the Q-learning algorithm (Watkins and Dayan, 1992), where the expected value Q*_i_*(*t*) of the selected option *i* in trial *t* is updated by the mismatch between the expected value and the actual outcome R(*t*), i.e., the prediction error *δ*_Q_(*t*), scaled by the learning rate *α*_Q_.


(1) $$Q_{i}(t+1)=Q_{i}(t)+\alpha *\delta_{Q}(t)$$

(2) $$\delta_{Q}\,\left(t\right)=R\,\left(t\right)-Q_{i}\,\left(t\right)$$

For all models, the probability *p* of selecting option *i* in trial *t* is modeled using a soft-max choice probability function:


(3) $$p_{i}\,\left(t\right)=\frac{e^{Q_{i}\,\left(t\right)*\beta_{Q}}}{\sum_{i}e^{Q_{i}\,\left(t\right)*\beta_{Q}}}$$

The decision weight β_*Q*_ determines how strongly a decision is affected by expected values, such that small values of β_*Q*_ allows for more stochastic decisions/exploration (Gershman, 2018; Wilson and Collins, 2019).

A popular derivation of the Q-learning algorithm separates the learning rates for feedbacks that are better or worse than expected (i.e., positive and negative prediction errors):


(4) $$Q_{i}\,\left(t+1\right)=Q_{i}\,\left(t\right)+\alpha_{\delta_{Q}+}*\delta_{Q}\,\left(t\right),i\,f\,\delta_{Q}\,\left(t\right)>0$$

(5) $$Q_{i}\,\left(t+1\right)=Q_{i}\,\left(t\right)+\alpha_{\delta_{Q}-}*\delta_{Q}\,\left(t\right),i\,f\,\delta_{Q}\,\left(t\right)<0$$

While these two models provide good fits to behavior in similar tasks (Frank et al., 2009; Aberg et al., 2015), neither model allows two interleaved tasks to interact (i.e., performance on one task is independent from the feedbacks received in other tasks). For this reason, we created a new set of models which allows the ARR *μ* to, respectively modulate the learning rate *α*, the decision weight *β_Q_*, and the prediction error signal *δ_Q_*:


(6) $$\alpha (t)=\alpha_{0}*(1+\mu (t))$$

(7) $$\beta_{Q}(t)=\beta_{0}*(1+\mu (t))$$

(8) $$\delta_{Q}(t)=R(t)-Q_{i}(t)+\mu (t)$$

The manipulations in Equations 6, 7, and 8, respectively allows larger values of μ to increase the learning rates, reduce exploration, and boost the prediction error signal.

Furthermore, the average reward may affect behavior in two separate ways. First, *via* the average accumulation of prediction errors (Eldar and Niv, 2015):


(9) $$\mu_{PE}(t+1)=\mu_{PE}(t)+\alpha_{\mu }*(\delta_{Q}(t)-\mu_{PE}(t))$$

Second, *via* the average accumulation of rewards (Aberg et al., 2020):


(10) $$\mu_{R}(t+1)=\mu_{R}(t)+\alpha_{\mu }*(R(t)-\mu_{R}(t))$$

To determine which of these two mechanisms that provide the best fit to behavior in our study, the two different ways of estimating μ, i.e., μ_*PE*_ and μ_*R*_, were included in different models.

Finally, based on the suggestion of one reviewer, we also included a model which tracks positive and negative outcomes in separate streams (Lin et al., 2020). Specifically, expected negative (*Q*_*negative*_) and positive (*Q*_*positive*_) outcomes are, respectively updated following negative (*R*_*negative*_) and positive (*R*_*positive*_) feedbacks:


(11) $$Q_{i,positive}(t+1)=\lambda_{+}*Q_{i,\,positive}(t)+\alpha *\delta_{Q,\,positive}(t)$$

(12) $$\delta_{Q,\,positive}(t)=w_{+}*R_{positive}(t)-Q_{i,\,positive}$$

(13) $$Q_{i,negative}(t+1)=\lambda_{-}*Q_{i,\,negative}(t)+\alpha *\delta_{Q,\,negative}(t)$$

(14) $$\delta_{Q\,,negative}(t)=w_{-}*R_{negative}(t)-Q_{i,\,negative}$$

This model allows the subjective weighting of negative and positive feedbacks *via* the free parameters *w*_−_ and *w*_+_, as well as the discounting of previous positive and negative outcomes *via* the free parameters. λ_+_ and λ_−_. Decisions are made by considering the combination of positive and negative expected outcomes:


(15) $$Q_{i,total}=Q_{i,positive}+Q_{i,negative}$$

Of note, some modifications of the original model were necessary to enable its fit to behavior in the present task. First, the original model presumes that both positive and negative feedbacks are presented in each trial, while in the present study only one feedback-type was presented. For this reason, when positive or negative feedbacks were received, *R*_*negative*_ or *R*_*positive*_ was, respectively set to 0. Second, in the original model the option with the largest total expected outcome [i.e., argmax(*Q*_*total*_)] is deterministically selected, an unrealistic assumption for human behavior in probabilistic reinforcement learning tasks. For this reason, decisions were modeled using the softmax probability function (Equation 3), with *Q*_*total*_ as the input. Importantly, this option still allows deterministic decision making (as would be indicated by fitting a very large β_*Q*_).

In total, nine different models were fitted to behavior:

• The canonical “ØØ-α” model, which is made up of Equations 1–3 with two free parameters: A learning rate α and a decision weight β_*Q*_.
• The “ØØ-αPE_+,–_” model, which is made up of Equations 2–5 with three free parameters: Two separate learning rates for negative and positive prediction errors α_δ_*Q*_−_, α_δ_*Q*_+_, and a decision weight β_*Q*_.
• The “ØØ-αR_+,–_” model, which is made up of Equations 3 and 11–14 with six free parameters: A learning rateα, two separate discount factors for negative and positive expected outcomes λ_−_, λ_+_, two separate weights for negative and positive outcomes w_−_, w_+_, and a decision weight β_*Q*_.
• The “FB-PE” model, which is made up of Equations 1, 3, 8, and 9 with three free parameters: A learning rate α, a learning rate for average reward α_μ_, and a decision weight β_*Q*_.
• The “α-PE” model, which is made up of Equations 1–3, 6, and 9 with three free parameters: A constant learning rate term (for when the average reward is zero) α_0_, a learning rate for average reward α_μ_, and a decision weight β_*Q*_.
• The “β-PE” model, which is made up of Equations 1–3, 7, and 9 with three free parameters: A learning rateα, a constant decision weight (for when the average reward is zero) β_0_, and a learning rate for average reward α_μ_.
• The “FB-R” model, which is made up of Equations 1,3, 8, and 10 with three free parameters: A learning rate α, a learning rate for average reward α_μ_, and a decision weight β_*Q*_.
• The “α-R” model, which is made up of Equations 1–3, 6, and 10 with three free parameters: A constant learning rate term (for when the average reward is zero) α_0_, a learning rate for average reward α_μ_, and a decision weight β_*Q*_.
• The “β-R” model, which is made up of Equations 1–3, 7, and 10 with three free parameters: A learning rateα, a constant decision weight (for when the average reward is zero) β_0_, and a learning rate for average reward α_μ_.

In summary, the “ØØ-α,” “ØØ-αPE_+,–_,” and the “ØØ-αR_+,–_ “models presume no impact of ARRs on performance. By contrast, the “FB-PE,” “α-PE,” and “β-PE” models allow the ARR to affect performance *via* an accumulation of prediction errors across tasks, while the “FB-R,” “α-R,” and “β-R” models allow the ARR to affect performance *via* an accumulation of feedbacks. Further, “FB-x,” “α-x,” and “β-x” models, respectively presume that the ARR affects performance by influencing the prediction error signal, the learning rate, and the decision weight.

All models were fitted to behavior and confronted using a hierarchical Bayesian inference (HBI) method (Piray et al., 2019). The HBI concurrently fits the free parameters and compares the considered models (while also correcting for differences in model complexity), something which allows constraining individual fits to group-level hierarchical priors. Additionally, the random effects approach used by the HBI calculates both group-level statistics and model evidence based on the posterior probability that the model explains each subject’s choice data. The HBI method provides fitted model parameters for each subject, as well as protected exceedance probabilities for each set of compared models. The exceedance probability estimates the probability that a model is the most frequent model to explain the observed behaviors, as compared to all other considered models (Rigoux et al., 2014). The *protected* exceedance probability (PXP) is more conservative, by taking into account the possibility that none of the compared models is supported by the data. To further demonstrate the robustness of the model selection procedure, we also report the model frequency, which is how often each model was determined to be the “best” model across participants.

To ensure that the parameters included in the selected model are meaningful, the values of the parameters used to simulate behaviors need to be successfully recovered when re-fitting the model to these simulated behaviors (Wilson and Collins, 2019). To confirm that this is the case, we randomly selected values of model parameters within the range of the fitted values obtained from the selected model, and generated the behavior of 1,000 virtual participants. Next, the selected model was re-fitted to the generated behaviors, and correlation coefficients were calculated between the generating and the recovered parameters. For a parameter to be meaningful, these correlations should be significantly positive.

## Results

### Behavioral results

#### Low average reward rate in the induction task reduces learning performance in the independent reference task

Learning curves collapsed across experiments are shown in Figure 1C, and for each experiment individually in Figure 1D. Learning performance for the reference task in each condition was defined as the average hit rate across the last third of the trials (i.e., trials 9–12). The average learning performance for the reference task in the different conditions collapsed across experiments are shown in Figure 1E, and for each experiment separately in Figure 1F.

To assess the impact of the ARR manipulation on learning performance in the reference tasks, and potential differences between experiments, the data from all participants were added to a mixed-factor ANOVA with between-subject Experiment (experiment 1–3) and Condition (inducer task with low, medium, and high reward probability). The results indicate a significant main effect of Condition [*F* (2,290) = 8.96, *p* < 0.001, $\eta_{p}^{2}$ 0.058, ANOVA], but no main effect of Experiment [*F* (2,145) = 2.62, *p* = 0.077, $\eta_{p}^{2}$ 0.035, ANOVA], nor interaction between Experiment and Condition [*F* (4,290) = 0.60, *p* = 0.665, $\eta_{p}^{2}$ 0.008, ANOVA]. For a full ANOVA table, see Table 1. The main effect of condition was due to significantly lower learning performance for the reference task in the low average reward condition, as compared to both the medium and the high average reward conditions [Figure 1E; low versus medium: *t* (147) = −4.063, 95% CI = −0.062, −0.022, *p* < 0.001, Cohen’s *d* = 0.334, two-tailed *t*-test; low versus high: *t* (147) = −3.236, 95% CI = −0.063, −0.015, *p* = 0.002, Cohen’s d = 0.266, two-tailed *t*-test]. By contrast, there was no difference in learning performance for the reference task in the medium and high average reward conditions [*t* (147) = 0.273, 95% CI = −0.017, 0.022, *p* = 0.785, Cohen’s d = 0.023, two-tailed *t*-test]. Notably, learning performance for the reference task in the low average reward condition was consistently lower in each experiment separately (for ANOVAs and *t*-tests, see Table 2).

**TABLE 1:** Mixed-effect ANOVA for the average hit rate as a function of Experiment (experiment 1, 2, and 3) and Condition (low, medium, and high reward probability).

	Sum of squares	*df*	Mean square	*F*	*P*-value	$\eta_{p}^{2}$
(Intercept)	274.92	1	274.92	5491.1	<0.001	
Experiment	0.262	2	0.131	2.615	0.077	0.035
Error	7.260	145	0.050			
Condition	0.156	2	0.078	8.959	<0.001	0.058
Experiment × condition	0.021	4	0.005	0.597	0.665	0.008
Error	2.528	290	0.009			

df: Degrees of Freedom.

F: F-statistic.

$\eta_{p}^{2}$: Partial eta-squared.

**TABLE 2:** Repeated measures ANOVAs for the average hit rate as a function of Condition (low, medium, and high reward probability) for each experiment separately.

	Sum of squares	*df*	Mean square	*F*/*t*	*P*-value	$\eta_{p}^{2}$/d
**Experiment 1**						
Repeated measures ANOVA						
(Intercept)	102.09	1	102.09	2465.4	<0.001	
Error	2.029	49	0.041			
Condition	0.065	2	0.033	3.926	0.023	0.080
Error	0.814	98	0.008			
Mean comparisons						
Low versus medium				−1.866	0.068	0.264
Low versus high				−2.390	0.021	0.338
Medium versus high				−1.181	0.243	0.167
**Experiment 2**						
Repeated measures ANOVA						
(Intercept)	111.58	1	111.58	2395.5	<0.001	
Error	2.655	57	0.047			
Condition	0.072	2	0.036	3.476	0.034	0.057
Error	1.185	114	0.010			
Mean comparisons						
Low versus medium				−2.480	0.016	0.326
Low versus high				−1.783	0.08	0.234
Medium versus high				0.538	0.593	0.071
**Experiment 3**						
Repeated measures ANOVA						
(Intercept)	69.76	1	69.76	1056.5	<0.001	
Error	2.575	39	0.066			
Condition	0.046	2	0.023	3.360	0.040	0.079
Error	0.529	78	0.007			
Mean comparisons						
Low versus medium				−2.881	0.006	0.456
Low versus high				−1.393	0.172	0.220
Medium versus High				1.091	0.282	0.172

df: Degrees of Freedom.

F/t: F-statistic/t-statistic.

$\eta_{p}^{2}$/d: Partial eta-squared/Cohen’s d.

In summary, supporting our predictions of an interaction between the inducer task and the reference task, we observed significantly lowered learning performance when the reference task was paired with an inducer task which provided low average reward. Furthermore, because these effects were observed in each of the three experiments, it cannot be attributed to differences in average reward magnitudes (tested in experiment 2) or increased salience attributed to negatively valued feedbacks (tested in experiment 3). Put simply, our data suggest interactions between two independent tasks performed in temporal proximity. Next, we turned to behavioral modeling to elucidate specific computational mechanism that may be touched by the ARR.

### Modeling results

Parameter fitting and model selection was performed concurrently using a HBI method (Piray et al., 2019). To select a model, we utilized the PXP, which estimates the likelihood of a model providing the best explanation of the observed behaviors, as compared to all other considered models, while also taking into account the possibility that none of the compared models is supported by the data.

**FIGURE 2:**
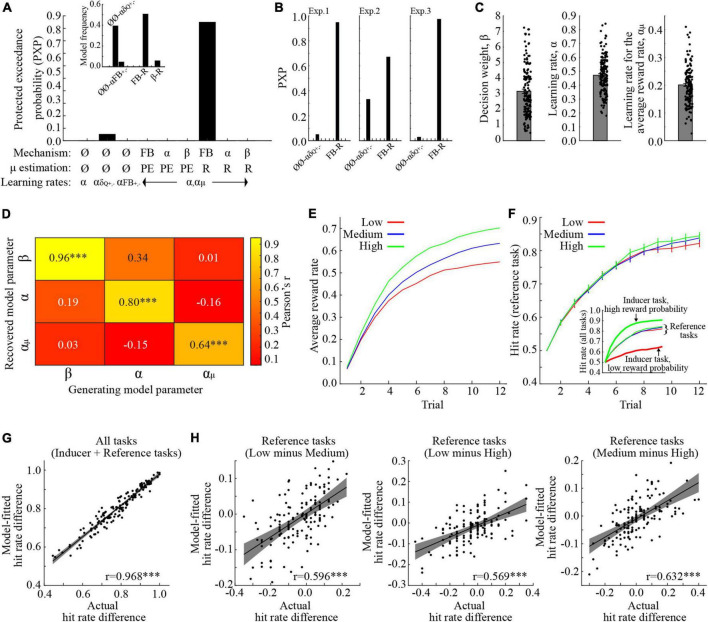
Behavioral modeling. **(A)** Protected exceedance probabilities (PXP) when model parameters were fitted across all experiments. The highest PXP was obtained for the FB-R model, where the average reward feedback influences the prediction error component of the model. The inset shows the model frequencies. **(B)** PXP’s when model parameters were fitted in each experiment separately. In all experiments, the FB-R model had the highest PXP. **(C)** The model parameters, when fitted across experiments. **(D)** Correlation coefficients between model parameters used to generate behavior, and their recovered counterparts. **(E)** The average reward rate for the reference tasks in the different conditions. **(F)** Model-fitted hit rates for the reference tasks, collapsed across the three experiments. The inset also shows performance for the inducer tasks with low- and high reward probabilities. Inducer/reference tasks are, respectively denoted by bold / narrow lines. **(G)** Correlation between actual- and model-fitted learning performance, averaged across all conditions and tasks. **(H)** Correlations between actual- and model-fitted differences in learning performance for the reference task in the different conditions. ***p < 0.001. All error bars indicate the standard error of the mean.

The PXPs for the tested models are shown in Figure 2A, with the largest PXP obtained for the “FB-R” model. In brief, the “FB-R” model presumes (i) that the ARR modulates the prediction error signal, and (ii) that manipulations of the ARR affects behavior *via* an accumulation of reward. Notably, the “FB-R” model obtained the highest PXP also when the analysis was repeated for each experiment separately (Figure 2B). For visualization purposes, the three parameters of the “FB-R” model are shown in Figure 2C. To demonstrate that these model parameters are meaningful (Wilson and Collins, 2019), we successfully recovered parameter values used to generate simulated behaviors (Figure 2D; see section “Materials and methods” for a description of this procedure).

One critical question is whether the “FB-R” model is capable of reproducing observed behaviors of interest. Before testing this, we first display the “FB-R” model’s estimate of average accumulated reward in each trial for the reference task in the different conditions (Figure 2E). As would be expected, the ARR increases when the inducer task provides higher reward probabilities. Model-fitted hit rates are shown in Figure 2F. To ensure that the selected model is capable of reproducing the same behavioral effects of interest, we entered model-fitted hit rates (averaged across trials 9–12) into the same mixed-effects ANOVA as for actual behavior. As with actual behavior, there was a significant main effect of Condition [*F* (2,290) = 7.217, *p* < 0.001, $\eta_{p}^{2}$ 0.047, ANOVA], and even though the model suggests a main effect of Experiment [*F* (2,145) = 3.847, *p* = 0.024, $\eta_{p}^{2}$ 0.050, ANOVA], there was no interaction between Experiment and Condition [*F* (4,290) = 0.170, *p* = 0.954, $\eta_{p}^{2}$ 0.002, ANOVA]. For a full ANOVA table and comparisons between model-fitted means, see Table 3.

**TABLE 3:** Repeated measures ANOVAs for the model-fitted average hit rate as a function of Condition (low, medium, and high reward probability).

	Sum of squares	*df*	Mean square	*F*/*t*	*P*-value	$\eta_{p}^{2}$/d
**Mixed-effects ANOVA**						
(Intercept)	292.13	1	292.13	7453.3	<0.001	
Experiment	0.302	2	0.151	3.847	0.024	0.050
Error	5.683	145	0.039			
Condition	0.036	2	0.018	7.217	<0.001	0.047
Experiment x Condition	0.002	4	<0.001	0.170	0.954	0.002
Error	0.720	290	0.003			
**Mean comparisons**						
Low versus medium				−2.185	0.031	0.180
Low versus high				−3.608	<0.001	0.297
Medium versus high				−1.771	0.079	0.146

df: Degrees of Freedom.

F/t: F-statistic/t-statistic.

$\eta_{p}^{2}$/d: Partial eta-squared/Cohen’s d.

These results indicate that the model captures group-level behaviors. However, a good model should also be able to capture inter-individual differences in behavior. For this reason, we first averaged learning performance across the last third of the trials across all conditions, and found a positive and significant correlation between actual- and model fitted learning performance (Figure 2G; Pearson’s *r* = 0.968, *p* < 0.001). Next, we correlated actual and model-fitted learning performances for our behavioral effects of interest, namely the differential hit rates for the reference task in the different conditions. All correlations were significant and positive [Low vs. Medium: Figure 2H, Pearson’s *r* = 0.596, *p* < 0.001; Low vs. High: Figure 2I, Pearson’s *r* = 0.569, *p* < 0.001; Medium vs. High: Figure 2J, Pearson’s *r* = 0.632, *p* < 0.001]. In other words, the “FB-R” model provides good fits to behavior, both on the group- and on the individual level.

In summary, we show that including the ARR in the model improves model fits. Moreover, the model with the most parsimonious fit presumes that ARR affects the prediction error signal *via* the accumulation of reward. These results extend the behavioral results by highlighting specific computational mechanism that is affected by manipulations of the ARR.

## Discussion

In three separate tasks, we observed reduced learning performance in a “reference” task when it was interleaved with an “inducer” task that provided a low probability of reward. These results support the notion that affective information obtained in one task lingers and affects performance in other, temporally proximal tasks, even when these tasks are independent. These results extend previous research showing that the ARR affects task performance across trials within the same task (Guitart-Masip et al., 2011; Rigoli et al., 2016; Aberg et al., 2017, 2020). As such, these studies may suggest a general role for the ARR in enabling interactions between temporally proximal events.

What cognitive mechanism(s) may be responsible for such interactions? It has been suggested that the ARR affects intrinsic motivation, e.g., low ARR reduces intrinsic motivation (Niv, 2007; Niv et al., 2007; Hamid et al., 2016). The evolutionary advantage of such a mechanism is that, for example, it enables the ability to preserve energy when resources (e.g., food/water) are scarce *via* a reduced motivation to exert effort. Additionally, it may provide a signal which regulates foraging behaviors (Constantino and Daw, 2015). The present study suggests that motivational transfer occurs not only across trials within the same task, but also across different tasks. An interesting topic for future studies is to what extent a low ARR contributes to exploratory decisions or task-switching.

The present study defines ARR as rewards per trial, but another option is to calculate it as rewards per time (i.e., the opportunity cost of time; Niv et al., 2007). While the two definitions are highly correlated in the present study (because trial durations are more or less constant), this distinction may be particularly important in the context of associative memory formation because large inter-trial intervals, which causes a reduction in the ARR per time, was found to improve associative memory performance (Lattal, 1999; Gallistel and Gibbon, 2000). While this result seemingly contrasts with our previous results (Aberg et al., 2017, 2020), we observe that large inter-trial intervals affords additional memory processes that might act to enhance memory performance, e.g., memory rehearsal (Reitich-Stolero and Paz, 2019). Yet, similarities and differences between the behavioral impact of average reward *per time* and *per trial* need to be addressed in future studies. For example, to test for motivational transfer of ARRs *per time*, the present task could be modified with an inducer task that applies different inter-trial intervals to manipulate the ARR per time.

Goal-directed motivation may depend on three factors, namely outcome controllability, outcome value, and effort cost (Grahek et al., 2019). In the present study, the cost of exerting effort was arguably similar across the different conditions. In addition, because all behavioral effects were similar across experiments with different outcome values, an effect which could be explained by the contextual scaling of available rewards (Palminteri et al., 2015), the impact of outcome values on motivation was negligible. By contrast, the different reward probabilities associated with the inducer task in the different conditions may have influenced the perceived outcome controllability (e.g., perceived controllability was large / small when reward probabilities were large / small). In other words, while the present study set out to test the following chain of events: “Average reward rate in inducer task -> Motivation -> Learning in reference task” path, we may actually have tested another chain of events, namely “Average reward rate in inducer task -> Perceived control in inducer task -> Motivation -> Learning in reference task.” Put simply, the reduced learning performance for the reference task in the condition with low reward probabilities may have been due to a transfer of reduced motivation induced by low perceived control in the inducer task. To test the motivational transfer of motivation induced by perceived control, an experiment could be conducted where the inducer task manipulates perceived control without altering the ARR. Such an experiment would test the chain “Perceived control inducer task -> Motivation -> Learning in independent task.”

Low perceived controllability has been associated with a variety of anxiety-related disorders, such as generalized anxiety disorder, post-traumatic stress disorder, panic disorder, social anxiety disorder, and obsessive-compulsive disorder (Gallagher et al., 2014), as well as depression (White, 1959; Abramson et al., 1989), and an increased vulnerability to develop a mental disorder (Barlow, 2000; White et al., 2006). Accordingly, the present study may inform psychopathology by showing that perceived control reduces intrinsic motivation, and how this may transfer to affect other independent behaviors. A potential flip-side of this research is that increasing the perception of control may also transfer and boost other motivated behaviors. Yet, future studies are clearly needed to investigate potential interactions between ARRs and perceived control, as well as the link between such interactions and inter-individual differences in personality traits and mental disorders.

The behavioral modeling revealed that the impact of ARRs on behavior is due to an alteration of the prediction error signal, rather than affecting learning rates or the tendency to make more exploratory decisions. This result resonates with our previous research showing that the ARR affected the neural correlates of feedback processing (Aberg et al., 2020), and suggestions that ARRs are coded in low tonic dopamine levels (Niv, 2007; Niv et al., 2007). However, recent evidence suggests that ARRs are associated with dopamine release in the nucleus accumbens (Hamid et al., 2016; Mohebi et al., 2019), but not with the activity of midbrain dopamine neurons (Mohebi et al., 2019). This seemingly surprising result, given that the nucleus accumbens receives dense projections from midbrain dopamine neurons (Ikemoto, 2007), indicates a complex relationship between ARRs, motivation, dopamine, and learning, which is still a hot topic for on-going research (for a recent insightful review, see Berke, 2018). Yet, because prediction errors are coded by midbrain dopamine neurons (Schultz, 2016) and the nucleus accumbens (Rutledge et al., 2010; Hart et al., 2014), it could be predicted that the impact of ARRs on reinforcement learning in the present tasks involves altered prediction error coding within these brain regions.

Behavioral models which included a factor that allowed the average rate to affect behavior consistently outperformed models which allowed no interaction between tasks. This result supports the notion of motivational transfers between tasks and trials, and therefore highlights the importance of including this interaction in future studies. For example, reinforcement learning tasks are sometimes presented in a block-wise fashion, but at other times in an inter-leaved fashion. Different models may therefore be needed to model behavior in these conditions in order to capture behavioral variability that can be attributed to motivational transfer between tasks. Another thing to consider is that while the ARR was here determined by the different feedbacks, it seems reasonable that an individual’s perceived performance may also contribute to the ARR. For example, intuition dictates that performing a difficult or effortful task would eventually lead to a drop in motivation, as compared to easier tasks, even without the presentation of explicit performance feedback. Interestingly, in visual perceptual learning, interleaving trials of a difficult task with trials of an easier tasks impedes learning, even in the presence of performance feedbacks (Aberg and Herzog, 2009, 2010). Future studies need to address the relationship between externally and internally generated performance feedbacks and motivational transfer *via* the ARR.

### Limitations

A first limitation is that both the inducer- and the reference task were reinforcement learning tasks, and therefore depended on the same neurocomputational mechanisms, e.g., prediction errors and the brain regions that code them. It therefore remains unknown whether our findings can be replicated using two different, e.g., two non-learning tasks or the combination of a learning- and a non-learning task. Even further, to what extent can ARRs obtained in a computerized game transfer and affect real-life motivated behaviors?

Second, we did not observe a difference in behavior between the conditions where the induced reward probability was medium and high, suggesting a non-linear relationship between ARRs and motivation. One explanation could be that the perceived difficulty/perceived controllability was similar for the medium and high average reward conditions (but different as compared to the low reward probability condition). Another option is that even if the difficulty/controllability is perceived as different, the motivational impact of medium and high reward probabilities could be similar. Supporting these notions, it was recently reported that the optimal rate of reward for binary classification learning was around 85% (Wilson et al., 2019), a value which is positioned rather in-between the medium (0.75) and the high (∼0.92) reward probabilities used in the present task. Yet, because we did not acquire any self-reported assessments about task-difficulties, this issue remains a topic for future studies.

## Data availability statement

The raw data supporting the conclusions of this article will be made available by the authors, without undue reservation.

## Ethics statement

The studies involving human participants were reviewed and approved by the Institutional Review Board (IRB) of the Weizmann Institute. Written informed consent was not provided because the experiment was conducted online, yet participation required providing informed consent by reading and agreeing to an online version of the consent form.

## Author contributions

KA and RP designed the experiment and wrote the manuscript. KA collected and analyzed the data. Both authors contributed to the article and approved the submitted version.
